# High-Dose Betahistine Improves Cognitive Function in Patients With Schizophrenia: A Randomized Double-Blind Placebo-Controlled Trial

**DOI:** 10.3389/fpsyt.2021.762656

**Published:** 2021-11-01

**Authors:** Yongqian Wang, Xufeng Huang, Hongzhen Fan, Huimei An, Ting Ma, Qi Zhang, Wenxuan Zhao, Yajun Yun, Wenshuang Yang, Xiaolu Zhang, Zhiren Wang, Fude Yang

**Affiliations:** ^1^Beijing HuiLongGuan Hospital, Peking University HuiLongGuan Clinical Medical School, Beijing, China; ^2^Illawarra Health and Medical Research Institute, School of Medicine, University of Wollongong, Wollongong, NSW, Australia

**Keywords:** schizophrenia, cognition, betahistine, histamine, H3-receptor antagonist

## Abstract

**Background:** There is currently no effective treatment for cognitive impairment associated with schizophrenia (CIAS). Recent studies have shown that increased histamine levels in the brain may help to improve CIAS symptoms. Betahistine is an H1-receptor agonist and H3-receptor antagonist. This study evaluated the effect of high-dose betahistine on cognitive function as well as its safety in Chinese Han patients with schizophrenia.

**Methods:** This randomized double-blind, placebo-controlled trial enrolled 89 patients with schizophrenia who were randomly administered betahistine (72 mg/d) or placebo for 12 weeks. At baseline and at 4, 8, and 12 weeks after commencing the intervention, we measured changes in cognitive function and clinical symptoms using the MATRICS Consensus Cognitive Battery (MCCB) and Positive and Negative Syndrome Scale (PANSS), respectively. Furthermore, we used the Treatment Emergent Symptom Scale (TESS) to assess the adverse effects of the patients' medications.

**Results:** Compared to the placebo group, the betahistine group showed significant improvements in the MCCB composite score after 12 weeks of treatment (*p* = 0.003) as well as improvements in MCCB verbal learning (*p* = 0.02) and visual learning (*p* = 0.001) domain scores. However, there were no significant improvements in the PANSS total scores or subscores (*p* > 0.05). Generally, high-dose betahistine treatment was considered safe in patients with schizophrenia.

**Conclusions:** Additional use of high-dose betahistine can effectively improve cognitive function but not psychiatric symptoms in patients with schizophrenia. Betahistine (72 mg/d) is well tolerated by Chinese Han patients with schizophrenia.

**Trial Registration:** chictr.org.cn, identifier: ChiCTR1900021078. http://www.chictr.org.cn/edit.aspx?pid=35484&htm=4

## Introduction

Schizophrenia encompasses a group of mental illnesses of unknown etiology. Patients often present with various symptoms affecting perception, thought, emotion, behavior, and cognition. At least 85% of the patients with schizophrenia have persistent and severe cognitive impairment associated with schizophrenia (CIAS), especially in attention, memory, and executive function. In the past, many studies have shown that cognitive function is the most critical factor affecting daily function and quality of life of patients with schizophrenia (1).

Several studies have demonstrated that second-generation antipsychotics (SGAs) are more effective at improving cognitive function in patients with schizophrenia than first-generation antipsychotics (FGAs). However, Marder et al. (2) noted that many patients treated with SGAs may still be affected by persistent cognitive impairment, and it is currently believed that FGAs and SGAs lead to limited improvement in the cognitive function of patients with schizophrenia (3).

The histaminergic system of the central nervous system (CNS) can be activated by a family of G protein-coupled receptors, including the H1, H2, H3, and H4 histamine receptors, thereby affecting sleep-wake cycles, attention, and metabolic homeostasis (4). Among these receptors, the H3 receptor is expressed on presynaptic membranes to regulate the release not only of histamine but also of acetylcholine (ACh), norepinephrine, dopamine, and serotonin, which are related to cognitive function and regulation of sleep-wake behavior (5). H3-receptor antagonists can promote the release of these neurotransmitters, thereby improving cognitive functions such as attention, working memory, and memory consolidation (6). Animal studies have also indicated that H3-receptor antagonists can enhance cognitive function by increasing histaminergic neurotransmission, thereby improving social recognition memory, spatial learning, and reference memory (7, 8). In clinical trials, H3-receptor antagonists have also demonstrated their ability to enhance cognition in different domains (9–11). These studies suggest that H3-receptor antagonists can be used to treat CIAS.

Betahistine is an H1-receptor agonist and H3-receptor antagonist. Clinically, it has been used for more than 40 years to treat Ménière's disease (MD). Betahistine enters the CNS and improves histaminergic neurotransmission (12). Although several studies have reported subsequent improvements in cognitive function (12–16), they have shown conflicting findings on the effects of betahistine on cognition. Insufficient betahistine doses and short intervention times may have been responsible for the inconsistent improvements in cognitive function observed in previous studies (17, 18). Ruitenbeek et al. (18) noted that addition of betahistine at 48 mg/d did not improve cognitive function in patients with schizophrenia. Researchers believe that after betahistine undergoes extensive first-pass metabolism, the blood concentration of betahistine cannot reach the level necessary to yield improvements in cognition (19). Other researchers have suggested that H3-receptor antagonists have dose-dependent brain cortical activation and wakening effects (20), indicating that the use of high-dose betahistine may improve cognitive function.

Therefore, the present study aimed to investigate the potential effects and safety of high-dose (72 mg/d) betahistine on cognitive function in patients with schizophrenia. To the best of our knowledge, this is the first randomized placebo-controlled study to evaluate the ability of betahistine to improve cognitive function in this population.

## Patients and Methods

### Study Design

This was a 12 week, randomized double-blind, placebo-controlled trial approved by the Ethics Committee of Beijing Huilongguan Hospital (No. 2018-47) and registered with the Chinese Clinical Trial Center (ChiCTR1900021078). After screening according to the medical record system of Beijing Huilongguan Hospital, an investigator met with patients who met the enrollment criteria described below. The investigator explained the content of the informed consent form to the patients in detail and answered their questions. All patients participated in the study voluntarily and signed the informed consent form. Betahistine hydrochloride (Xinxiang LepuHengjiuyuan Pharmaceutical Co., Ltd, Henan, China) was used in this study; the specification was 4 mg/tablet. Patients in the betahistine group took 72 mg of betahistine hydrochloride per day (24 mg *t.i.d*). Patients in the placebo group took placebo at the same frequency and dose as the betahistine group. The compliance of patients with medication was the responsibility of the doctor in charge and the nurse who was mainly responsible for medication use. The patient took the medicine daily under the supervision of the doctor in charge and medication nurse. We also calculated the patient's medication compliance based on the number of final recalled drugs.

### Participants

A total of 109 inpatients with schizophrenia were recruited from Beijing Hui-Long-Guan hospital between January 2019 and January 2020. All patients met the following inclusion criteria: (1) inpatients status and diagnosis of schizophrenia in accordance with the International Classification of Diseases (ICD)-10 criteria, (2) aged 20–59 years, (3) Han nationality, (4) right-handedness, (5) currently use of SGAs at a stable dose over the preceding 6 weeks, (6) education ≥6 years, and (7) stable weight (±5%) for at least 6 months. The exclusion criteria were as follows: (1) use of cognition-enhancing drugs or other investigational agents during the study, (2) electroconvulsive treatment within the previous 2 months, (3) severe or unstable physical diseases, (4) history of alcohol or drug abuse within the previous 12 months, (5) history of severe drug allergy or allergic predisposition, (6) pregnancy or lactation, and (7) betahistine contraindications such as active or a history of peptic ulcer and/or bronchial asthma.

### Randomization and Blinding

The randomization table was generated using a computer by the researcher who designed this study. An independent statistician generated a random allocation list using block randomization with variable block sizes (*n* = 4), which was used to assign participants. The pharmacist participating in the study placed the random numbers in ordinary airtight envelopes marked with the patient numbers. The envelope was kept in the pharmacy and was only opened by the pharmacist when randomizing the study participants. Individuals directly involved in the research have no access to these envelopes. Patients in the placebo group were given the same number of pills and followed the same medication schedule as the betahistine group. To ensure the implementation of the blinding method, medications for both the betahistine group and placebo group were placed in opaque white bottles with the same appearance. Both the patient and the evaluator of the study were blinded to patient grouping. It should be noted that, according to previous studies, betahistine doses of 72 mg/d (or higher) will not cause serious side effects (21). Therefore, the participants in this trial were blinded to the medications they were taking (betahistine or placebo).

### Outcome Measures

The primary outcome of this study was cognitive function in patients with schizophrenia. The assessment of cognitive function was carried out as per the Measurement and Treatment to Improve Cognition in Schizophrenia (MATRICS) initiative. We used the MATRICS Consensus Cognitive Battery (MCCB), which includes the following seven major cognition domains: speed of processing, attention/vigilance, working memory, verbal memory, visual learning, reasoning and problem solving, and social cognition (22). Cognitive function was assessed at baseline and 4, 8, and 12 weeks after the drug intervention. MCCB scores were converted into T-scores based on age and sex. High T-scores are considered indicative of high-level cognitive ability. All researchers who conducted the MCCB testing had been trained to do the same.

At baseline and 4, 8, and 12 weeks after the intervention, we also evaluated the patient's psychiatric symptoms. The Positive and Negative Syndrome Scale (PANSS) was used to evaluate the severity of psychiatric symptoms (23). The PANSS scale consists of seven positive symptom scores, seven negative symptom scores, and 16 general psychopathological symptom scores. High PANSS scores reflect severe psychiatric symptoms. The investigators completed consistency training for these scales (intraclass correlation coefficient of >0.8). Furthermore, we used the Treatment Emergent Symptom Scale to evaluate adverse treatment effects (24).

### Sample Size

According to the results of our previous preliminary experiments, betahistine improved cognitive function with an average of 6.7 points and a standard deviation (SD) of 6 points in the MCCB composite score. Previous studies have shown that the average 3 month improvement in the MCCB composite score of patients with schizophrenia not taking betahistine was 2.5 (α = 0.05, power = 0.8) (25). Based on our sample size calculation (see Supplementary Material) and considering a dropout rate of ~20%, our study required at least 42 people in each group.

### Statistical Analyses

Data analysis was based on intent-to-treat principles. Missing observations were imputed by using the most recent previous observation (the last observation carried forward). Descriptive statistics were used to summarize the baseline characteristics of the participants using the mean and standard deviation (SD) or frequency (proportion), as appropriate. The baseline characteristics of participants were compared according to intervention, using the two-sample *t*-test for numerical variables and the chi-square test for categorical variables. The differences in the total and subscale scores of the MCCB and PANSS from baseline to week 12 were compared using repeated-measures analysis of variance (ANOVA). The inter-subject factor was the group (betahistine group or placebo group), whereas the intra-subject factor was the time of measurement (baseline and 4, 8, and 12 weeks after the drug intervention). The analyses were performed for each measurement variable, for both the inter- and intra-subject factors, and for interactions in the same group. Cohen's d was calculated to estimate the effect size of the mean intra-group differences in the betahistine and placebo groups. All the analyses were performed using the SPSS 21 package, and bilateral significance tests were also performed, with a significance level of 5%.

## Results

### Patient Characteristics

**Figure 1:**
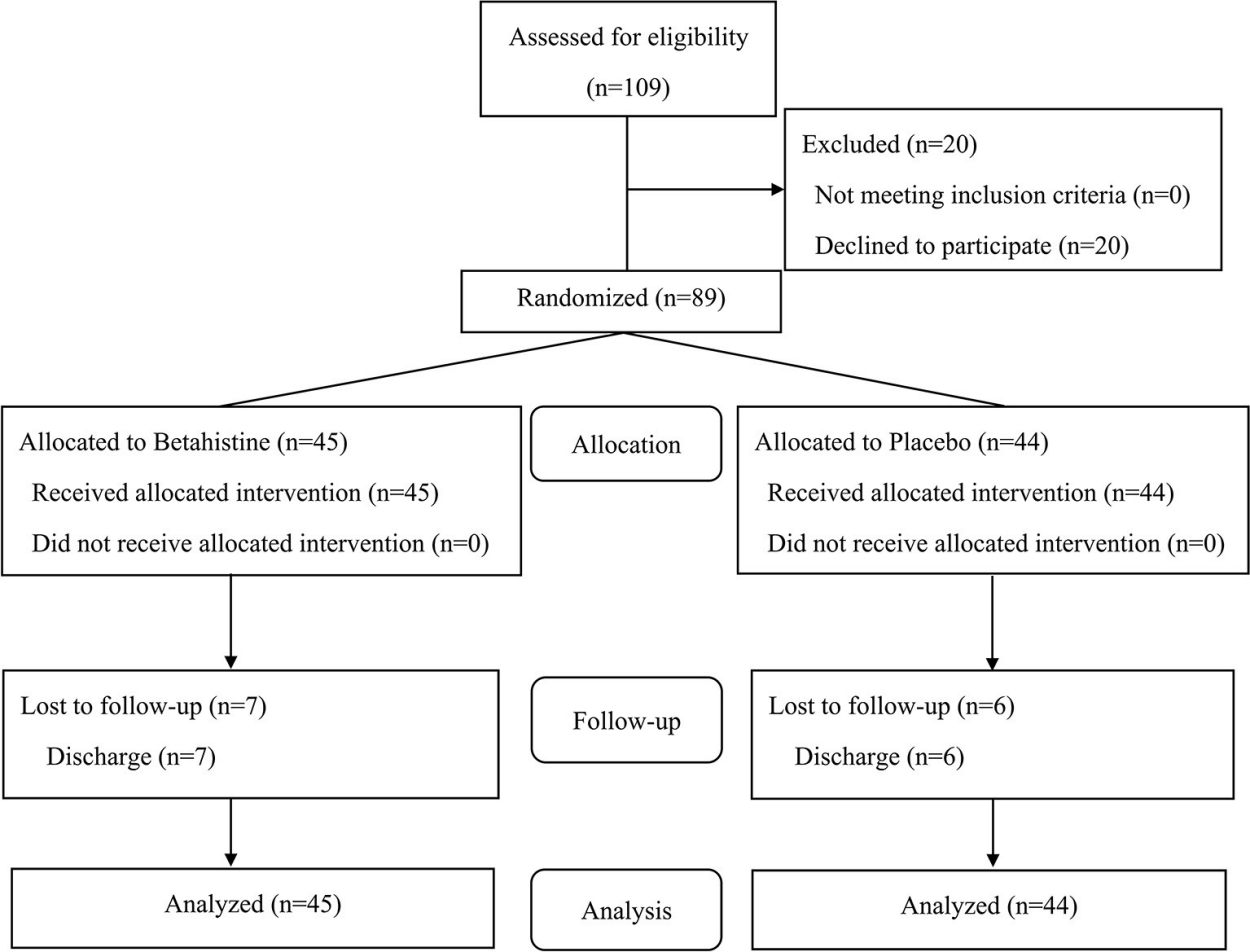
CONSORT flow chart for the present study.

We selected patients from Beijing Huilongguan Hospital who met the enrollment criteria between January 2019 and July 2020. A total of 109 patients signed the informed consent form, and 20 patients refused to participate in the study before the test began. Thus, 89 patients participated in the randomization. There were 45 patients in the betahistine group and 44 patients in the placebo group. A total of 13 patients dropped out during the trial (dropout rate: 14.61%). In the betahistine group, 7 were discharged due to discharge (2 were discharged between baseline and 1 month after the intervention, and 5 were discharged 2 months after the intervention). In the placebo group, 6 people were discharged due to discharge (2 were discharged between baseline and 1 month after the intervention, and 4 were discharged 2 months after the intervention). The patients in the betahistine and placebo group withdrew from the study because they were all discharged (Figure 1).

The demographic characteristics of the study population and ratings during screening for each arm of the study are shown in Table 1. There were no significant differences between the betahistine and placebo groups in terms of age (47.40 ± 8.24 and 47.02 ± 9.58 years, respectively), sex (male: 27 and 27, respectively), education (12.96 ± 2.77 and 12.93 ± 3.74 years, respectively), diagnosis (paranoid schizophrenia/residual schizophrenia/undifferentiated schizophrenia:11/32/2 and 10/33/1, respectively), course of illness (23.40 ± 10.58 and 23.02 ± 11.06 years, respectively), course of treatment (21.08 ± 10.58 and 19.86 ± 12.05 years, respectively), smoking status (Yes/No: 14/ 31 and 11/ 33, respectively), chlorpromazine equivalent dose (350.06 ± 128.06 and 303.98 ± 188.71 mg, respectively), treatment with anticholinergic agents (Yes/No: 11/34 and 7/37, respectively), body mass index (25.33 ± 3.43 and 25.26 ± 4.87, respectively), waist circumference (93.53 ± 9.20 and 92.10 ± 15.32 cm, respectively), or hip circumference (99.24 ± 7.35 and 99.42 ± 11.66 cm, respectively). At baseline, there were also no significant differences in cognitive function or the severity of symptoms between the groups.

**Table 1:** Baseline characteristics of participants by intervention.

**Characteristic**	**Betahistine group**	**Placebo group**	***p*-value^**a**^**
	**(*n* = 45)**	**(*n* = 44)**	
Age, mean (SD), y	47.40 (8.24)	47.02 (9.58)	0.925
Male, no. (%)	27 (60.00)	27 (61.36)	0.89
Education, mean (SD), y	12.96 (2.77)	12.93 (3.74)	0.97
Diagnosis (p/u/r), (*n*)	11/32/2	10/33/1	0.83
Course of illness, mean (SD), y	23.40 (10.58)	23.02 (11.06)	0.87
Course of treatment, mean (SD), y	21.08 (10.58)	19.86 (12.05)	0.61
Smoking status (Y/N)	14/31	11/33	0.52
Years of smoking, mean (SD), y	15.14 (9.62)	20.91 (8.32)	0.13
Daily number of cigarettes, mean (SD), *n*	17.86 (13.96)	18.00 (15.07)	0.98
CPZ equivalent dose, mean (SD), (mg/d)	350.06 (128.06)	303.98 (177.71)	0.16
Anticholinergic agent (Y/N)	11/34	7/37	0.32
BMI, mean (SD)	25.33 (3.43)	25.26 (4.87)	0.94
Waistline, mean (SD), cm	93.53 (9.20)	92.10 (15.32)	0.87
Hipline, mean (SD), cm	99.24 (7.35)	99.42 (11.66)	0.81

a *The p-value was determined using either the two-sample t-test or the chi-square independent test. BMI, body mass index; SD, standard deviation; p, paranoid schizophrenia; r, residual schizophrenia; u, undifferentiated schizophrenia; CPZ, chlorpromazine*.

### Cognitive Effects

**Figure 2:**
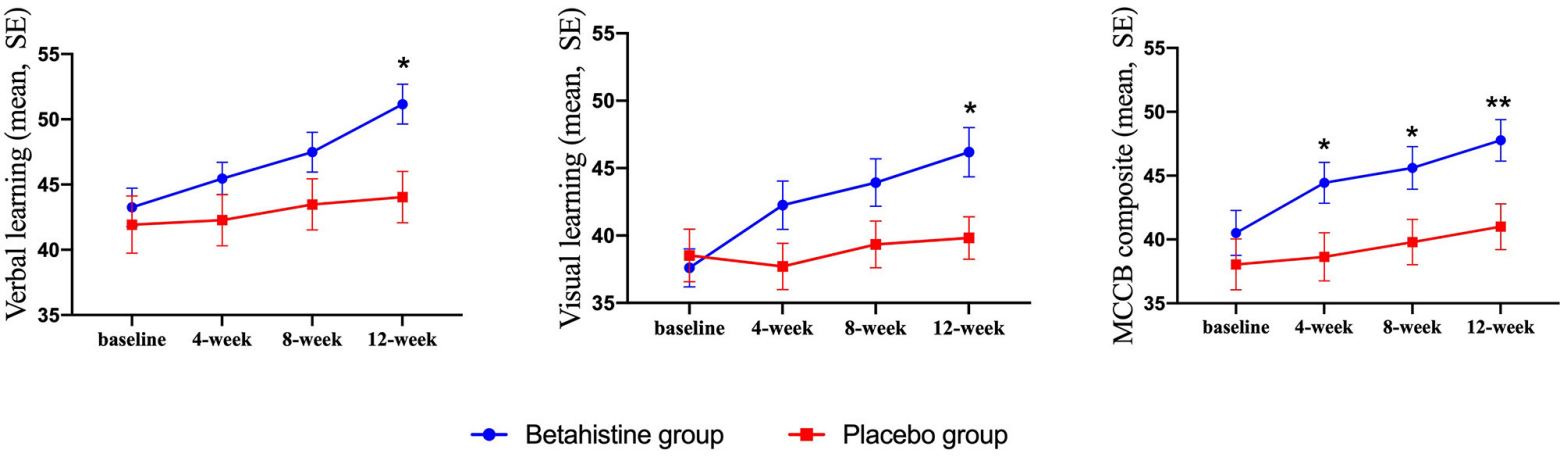
Drug effect on cognitive function scores at each visit. Single asterisk (*) indicates a significant effect when compared with placebo at the same visit with a *p*-value of < 0.05. Double asterisks indicate a significant effect when compared with placebo at the same visit with a *p*-value of < 0.01.

Changes in cognitive function (MCCB) in the betahistine and placebo groups at each visit over the 12 week study period are shown in Table 2. There were time-group effects for verbal learning (*F* = 3.44, *p* = 0.02, Cohen's d = 0.63), visual learning (*F* = 6.07, *p* = 0.001, Cohen's d = 0.81), and MCCB composite score (*F* = 5.32, *p* = 0.003, Cohen's d = 0.68) in favor of the betahistine group, but not for reasoning and problem-solving (*F* = 0.86, *p* = 0.46), social cognition (*F* = 0.91, *p* = 0.43), attention/vigilance (*F* = 2.23, *p* = 0.10), speed of processing (*F* = 2.27, *p* = 0.10), or working memory (*F* = 2.39, *p* = 0.08). Aspects of cognitive function with time-group effects and changes in those aspects from baseline to each subsequent visit are shown in Figure 2. The betahistine group exhibited a significant difference in verbal learning and visual learning as compared with the placebo group after 12 weeks of treatment (*p* = 0.002, *p* = 0.008, respectively). The MCCB composite scores were statistically different between the betahistine group and placebo group at weeks 4, 8, 12 (*p* = 0.042, *p* = 0.032, *p* = 0.008, respectively).

**Table 2:** MCCB total and subscale scores at baseline and weeks 4, 8, and 12 in the betahistine and placebo groups.

	**Betahistine group**	**Placebo group**	**Time F (*p*-value)**	**Group F (*p*-value)**	Group*Time F (*p*-value)
**Speed of processing**			20.33 (<0.01)	3.19 (0.08)	2.27 (0.10)
Baseline	42.63 ± 10.95	40.66 ± 10.33			
Week 4	46.05 ± 10.67	41.61 ± 10.82			
Week 8	46.40 ± 10.32	41.93 ± 10.48			
Week 12	48.56 ± 10.57	43.98 ± 10.00			
**Attention/Vigilance**			2.25 (0.10)	3.43 (0.07)	2.23 (0.10)
Baseline	44.30 ± 11.21	42.27 ± 11.71			
Week 4	47.74 ± 10.70	42.41 ± 10.65			
Week 8	46.47 ± 10.15	41.95 ± 11.89			
Week 12	46.42 ± 10.66	41.64 ± 11.46			
**Working memory**			17.32 (<0.01)	3.3 (0.10)	2.39 (0.08)
Baseline	45.35 ± 14.59	43.70 ± 12.51			
Week 4	51.37 ± 12.26	45.52 ± 13.64			
Week 8	52.05 ± 11.48	46.93 ± 12.40			
Week 12	53.09 ± 9.84	47.73 ± 12.26			
**Verbal learning**			11.05 (<0.01)	3.08 (0.08)	**3.44 (0.02)**
Baseline	43.26 ± 9.51	41.93 ± 14.41			
Week 4	45.47 ± 8.07	42.27 ± 12.86			
Week 8	47.49 ± 9.89	43.48 ± 12.84			
Week 12	51.16 ± 9.89	44.05 ± 12.91			
**Visual learning**			11.05 (<0.01)	2.81 (0.10)	**6.07 (0.001)**
Baseline	37.60 ± 9.11	38.52 ± 12.81			
Week 4	42.26 ± 11.60	37.70 ± 11.28			
Week 8	43.93 ± 11.38	39.34 ± 11.33			
Week 12	46.19 ± 11.8	39.82 ± 10.35			
**Reasoning and problem solving**			11.92 (<0.01)	6.19 (0.01)	0.86 (0.46)
Baseline	46.35 ± 12.95	41.52 ± 10.99			
Week 4	46.77 ± 10.37	39.95 ± 12.38			
Week 8	48.42 ± 10.7	43.55 ± 9.86			
Week 12	49.84 ± 10.77	45.5 ± 10.48			
**Social cognition**			0.17 (0.90)	0.73 (0.40)	0.91 (0.43)
Baseline	41.49 ± 10.31	40.48 ± 11.78			
Week 4	41.63 ± 12.07	41.50 ± 10.6			
Week 8	42.58 ± 12.13	39.77 ± 10.36			
Week 12	43.19 ± 12.26	40.09 ± 10.08			
**MCCB total score**			27.5 (<0.01)	4.72 (0.03)	**5.32 (0.003)**
Baseline	40.51 ± 11.40	38.05 ± 13.07			
Week 4	44.44 ± 10.38	38.64 ± 12.37			
Week 8	45.60 ± 10.79	39.80 ± 11.65			
Week 12	47.77 ± 10.51	41.00 ± 11.75			

MCCB, MATRICS Consensus Cognitive Battery; Bold value means Group*Time p < 0.05.

### Psychiatric Symptoms

The changes in schizophrenia symptoms after 12 weeks of intervention are shown in Table 3. There were no time-group effects for positive symptom scores (*F* = 1.59, *p* = 0.20), negative symptom scores (*F* = 1.60, *p* = 0.20), general scores (*F* = 0.85, *p* = 0.40), or total scores (*F* = 1.82, *p* = 0.16) in favor of the betahistine group.

**Table 3:** PANSS total and subscale scores at baseline, week 4, week 8, and week 12 in the betahistine and placebo groups.

	**Betahistine group**	**Placebo group**	**Time F (*p*-value)**	**Group F (*p*-value)**	Group*Time F (*p*-value)
**PANSS total score**			7.51 (<0.01)	0.28 (0.60)	1.82 (0.16)
Baseline	74.77 ± 14.48	75.34 ± 13.56			
Week 4	72.79 ± 13.17	75.27 ± 13.23			
Week 8	72.84 ± 12.22	74.41 ± 13.51			
Week 12	72.53 ± 11.96	73.82 ± 13.12			
**Positive subscale score**			2.11 (0.11)	2.18 (0.14)	1.59 (0.20)
Baseline	16.98 ± 5.20	18.16 ± 5.06			
Week 4	17.00 ± 4.79	18.89 ± 4.92			
Week 8	16.98 ± 4.74	18.75 ± 5.07			
Week 12	16.91 ± 4.84	18.20 ± 4.71			
**Negative subscale score**			28.41 (<0.01)	0.22 (0.64)	1.60 (0.20)
Baseline	23.23 ± 8.08	23.30 ± 7.32			
Week 4	21.93 ± 7.26	22.86 ± 6.75			
Week 8	21.63 ± 6.83	22.52 ± 6.79			
Week 12	20.63 ± 6.31	21.55 ± 6.69			
**General subscale score**			5.45 (0.01)	0.30 (0.59)	0.85 (0.40)
Baseline	34.56 ± 7.07	33.89 ± 6.45			
Week 4	33.86 ± 6.84	33.52 ± 6.59			
Week 8	34.23 ± 6.19	33.14 ± 6.68			
Week 12	35.00 ± 6.32	34.07 ± 6.40			

### Adverse Effects

No serious adverse events occurred in the trial (details see Supplementary Table 1). There were no differences in side-effects between the two groups in terms of any rated symptom, and there were no betahistine-related side effects.

## Discussion

This study aimed to examine the effects of betahistine on cognitive function in patients with schizophrenia. It is the first randomized placebo-controlled study on the use of high-dose betahistine to treat cognitive impairment in these patients. Simultaneously, this is also the first clinical study to assess the safety of high-dose betahistine (72 mg/d) in a Chinese Han population with schizophrenia. Our analysis indicated that high-dose betahistine can improve cognitive function across various domains in patients with schizophrenia, but not positive or negative symptoms. Our findings also suggest that high-dose betahistine (72 mg/d) is safe in the Chinese Han population.

The MCCB scores were significantly higher in the betahistine group than in the placebo group from weeks 4 to 12. This result suggests that high-dose betahistine (72 mg/d) can significantly improve the cognition of patients who require long-term hospitalization. In the betahistine group, the MCCB composite score increased by 7.26 points after 3 months of treatment. In clinical terms, we believe that an increase in the MCCB composite score > 5 points has certain clinical significance. Well before the development of antipsychotics, some researchers suggested that histamine levels are associated with schizophrenia (26). Recently, some studies have shown that H3-receptor antagonists can improve cognitive function (5, 8, 20) by influencing receptors expressed in several brain regions related to cognition (17). Not only do H3 receptor antagonists increase histamine levels by blocking presynaptic membrane H3 receptors, but H3 receptors also act as heteroreceptors to regulate the release of several neurotransmitters relevant to cognition, such as ACh, dopamine, norepinephrine, and serotonin (10, 27). Studies have demonstrated that H3-receptor antagonists, such as BF2.649 and GSK189254, improve cognition by increasing ACh release in the frontal cortex and/or dorsal hippocampus, which are considered important for cognitive function (20, 28). In addition to increased ACh release, the histamine-receptor antagonist ABT-239 leads to elevated dopamine concentrations in the rat prefrontal cortex (29). Similar results were reported by Ligneau et al. and Medhurst et al. (20, 28). Noradrenergic neurotransmission within the CNS plays an important role in attentional processing and affective behaviors, which is highly regulated through norepinephrine release in the cortical and hippocampal regions from the axon terminals of neurons located in the locus coeruleus (5). Accordingly, rat studies have shown that H3 antagonists increase basal norepinephrine levels in the cingulate cortex and improve cognitive function (28). Given that serotonergic neurons project axons throughout the cortical and hippocampal forebrain regions where H3 receptors are located (5), H3 receptor-mediated serotonin release may also cause changes in cognitive function.

Our findings indicated that betahistine treatment significantly improved verbal learning, visual learning, and MCCB composite scores. Previous studies have reported that verbal learning is related to dopamine levels in the brain (30–32). Takahashi et al. (33) further demonstrated that the level of dopamine in the hippocampus is related to verbal memory. Additionally, dopamine affects visual function, including contrast sensitivity, visual memory, and electroretinographic responses (34). The mechanism by which betahistine improves these three MCCB scores may involve increased levels of neurotransmitters in the brain, especially dopamine.

In accordance with previous findings, the betahistine group did not show significant improvements in the PANSS scores relative to the placebo group. Michael et al. demonstrated that MK-0249, anH3R antagonist, did not significantly improve PANSS scores in patients with schizophrenia (35). Similarly, researchers investigating the use of another H3 receptor antagonist (GSK239512) in stable outpatients with schizophrenia did not report significant improvements in the PANSS scores in the drug intervention group (28). It should be noted that in this study, betahistine was added to existing antipsychotic treatment, which may have blunted its potential effects on schizophrenia symptoms (36).

The present study is the first to investigate the use of high-dose betahistine in Chinese Han patients with schizophrenia. Our analysis revealed that this drug exhibited a good safety profile, consistent with the results of previous studies comprising patients with schizophrenia and MD. After adding 144 mg/d of betahistine or placebo to the treatment regimen in 48 patients, Barak et al. (37) observed good tolerance and compliance with high-dose betahistine in the intervention group, and no serious adverse events were reported. Some studies also reported that the use of betahistine at doses of 288–480 mg/d does not lead to adverse effects (38).

This study had several limitations. First, this study only used a single dose of betahistine for patients with schizophrenia, and we did not include a multiple-dose control group. Previous studies have suggested that different doses of H3-receptor antagonists exhibit different abilities in crossing the blood-brain barrier, which may lead to different effects on cognitive function (19). Second, the patients with schizophrenia included in this study had a long treatment period, and their symptoms were relatively stable, which may explain the lack of significant improvement in their symptoms. Future studies can add multiple dose groups to observe whether different doses improve cognitive function to different degrees. Moreover, future studies may wish to examine the effect of betahistine on psychiatric symptoms in patients with first-episode schizophrenia.

## Conclusions

In general, the present study demonstrated that high-dose betahistine can improve cognitive function in Chinese Han patients with schizophrenia, but not psychiatric symptoms. Our findings also indicate that such treatment is well tolerated in this population.

## Data Availability Statement

The datasets presented in this article are currently not readily available due to privacy concerns. Requests to access the datasets should be directed to 799722491@qq.com.

## Ethics Statement

The studies involving human participants were reviewed and approved by Ethics Committee of Beijing Huilongguan Hospital (No. 2018-47). The patients/participants provided their written informed consent to participate in this study.

## Author Contributions

YW, FY, and ZW designed this study. YW wrote this manuscript. TM, QZ, WZ, and YY assisted in this research. HF and HA assisted in data analysis. XH assisted in revising the paper. All authors reviewed and helped revise the manuscript.

## Funding

This research was supported by the National Key R&D Program of China (2016YFC1306804), the Beijing Hospital Authority Ascent Plan (DFL20182001), and Beijing Hospitals Authority Clinical Medicine Development of Special Funding (XMLX202130). The funding sources had no involvement in the study design or conduct or in the collection, analysis, and interpretation of data.

## Conflict of Interest

The authors declare that the research was conducted in the absence of any commercial or financial relationships that could be construed as a potential conflict of interest.

## Publisher's Note

All claims expressed in this article are solely those of the authors and do not necessarily represent those of their affiliated organizations, or those of the publisher, the editors and the reviewers. Any product that may be evaluated in this article, or claim that may be made by its manufacturer, is not guaranteed or endorsed by the publisher.

## References

[1] Barch DM Sheffield JM. Cognitive impairments in psychotic disorders: common mechanisms and measurement. World Psychiatry. (2014) 13:224–32. 10.1002/wps.20145 25273286 PMC4219054

[2] Marder SR Fenton W. Measurement and treatment research to improve cognition in schizophrenia: NIMH MATRICS initiative to support the development of agents for improving cognition in schizophrenia. Schizophr Res. (2004) 72:5–9. 10.1016/j.schres.2004.09.010 15531402

[3] Tandon R Nasrallah HA Keshavan MS. Schizophrenia, “just the facts” 5. treatment and prevention Past, present, and future. Schizophr Res. (2010) 122:1–23. 10.1016/j.schres.2010.05.025 20655178

[4] Leurs R Vischer HF Wijtmans M de Esch IJP. En route to new blockbuster anti-histamines: surveying the offspring of the expanding histamine receptor family. Trends Pharmacol Sci. (2011) 32:250–7. 10.1016/j.tips.2011.02.004 21414671

[5] Esbenshade TA Browman KE Bitner RS Strakhova M Cowart MD Brioni JD. The histamine H3 receptor: an attractive target for the treatment of cognitive disorders. Br J Pharmacol. (2008) 154:1166–81. 10.1038/bjp.2008.147 18469850 PMC2483387

[6] Brioni JD Esbenshade TA Garrison TR Bitner SR Cowart MD. Discovery of histamine H3 antagonists for the treatment of cognitive disorders and Alzheimer's disease. J Pharmacol Exp Ther. (2011) 336:38–46. 10.1124/jpet.110.166876 20864505

[7] Esbenshade TA Browman KE Miller TR Krueger KM Komater-Roderwald V Zhang M. Pharmacological properties and procognitive effects of ABT-288, a potent and selective histamine H3 receptor antagonist. J Pharmacol Exp Ther. (2012) 343:233–45. 10.1124/jpet.112.194126 22815533

[8] Mahmood D Khanam R Pillai KK Akhtar M. Protective effects of histamine H3-receptor ligands in schizophrenic behaviors in experimental models. Pharmacol Rep. (2012) 64:191–204. 10.1016/S1734-1140(12)70746-6 22580536

[9] Cho W Maruff P Connell J Gargano C Calder N Doran S. Additive effects of a cholinesterase inhibitor and a histamine inverse agonist on scopolamine deficits in humans. Psychopharmacology. (2011) 218:513–24. 10.1007/s00213-011-2344-y 21644059

[10] Haig GM Bain E Robieson W Othman AA Baker J Lenz RA. randomized trial of the efficacy and safety of the H3 antagonist ABT-288 in cognitive impairment associated with schizophrenia. Schizophr Bull. (2014) 40:1433–42. 10.1093/schbul/sbt240 24516190 PMC4193706

[11] Jucaite A Takano A Boström E Jostell K-G Stenkrona P Halldin C. AZD5213: a novel histamine H3 receptor antagonist permitting high daytime and low nocturnal H3 receptor occupancy, a PET study in human subjects. Int J Neuropsychopharmacol. (2013) 16:1231–9. 10.1017/S1461145712001411 23217964

[12] Tighilet B Trottier S Lacour M. Dose- and duration-dependent effects of betahistine dihydrochloride treatment on histamine turnover in the cat. Eur J Pharmacol. (2005) 523:54–63. 10.1016/j.ejphar.2005.09.017 16226741

[13] Baqui Q Banjar WMA Gazzazz J Hou RH Langley RW Szabadi E. Comparison of betahistine and diphenhydramine on alertness and autonomic functions in healthy volunteers. J Psychopharmacol. (2008) 22:A33. 10.1177/0269881106071022 17092978

[14] Gordon CR Doweck I Nachum Z Gonen A Spitzer O Shupak A. Evaluation of betahistine for the prevention of seasickness: effect on vestibular function, psychomotor performance and efficacy at sea. J Vestib Res. (2003) 13:103–11. 10.3233/VES-2003-132-305 14757913

[15] Szabadi E Banqui Q Banjar WMA Gazzaz J Langley RW Bradshaw CM. Interactions between modafinil and betahistine on alertness and automatic functions in healthy volunteers. J Psychopharmacol. (2009) 23:A60.

[16] Tighilet B Trottier S Mourre C Chotard C Lacour M. Betahistine dihydrochloride interaction with the histaminergic system in the cat: neurochemical and molecular mechanisms. Eur J Pharmacol. (2002) 446:63–73. 10.1016/S0014-2999(02)01795-8 12098586

[17] Nomura H Mizuta H Norimoto H Masuda F Miura Y Kubo A. Central histamine boosts perirhinal cortex activity and restores forgotten object memories. Biol Psychiatry. (2019) 86:230–9. 10.1016/j.biopsych.2018.11.009 30635130

[18] van Ruitenbeek P Mehta MA. Potential enhancing effects of histamine H1 agonism/H3 antagonism on working memory assessed by performance and bold response in healthy volunteers. Br J Pharmacol. (2013) 170:144–55. 10.1111/bph.12184 23517178 PMC3764856

[19] Chen XY Zhong DF Duan JL Yan BX. LC-MS-MS. analysis of 2-pyridylacetic acid, a major metabolite of betahistine: application to a pharmacokinetic study in healthy volunteers. Xenobiotica. (2003) 33:1261–71. 10.1080/716689336 14765546

[20] Ligneau X Landais L Perrin D Piriou J Uguen M Denis E. et al. Brain histamine and schizophrenia: potential therapeutic applications of H3-receptor inverse agonists studied with BF2649. Biochem Pharmacol. (2007) 73:1215–24. 10.1016/j.bcp.2007.01.023 17343831

[21] Jeck-Thole S Wagner W. Betahistine: A retrospective synopsis of safety data. Drug Safety. (2006) 29:1049–59. 10.2165/00002018-200629110-00004 17061910

[22] Green MF Nuechterlein KH. The MATRICS initiative: developing a consensus cognitive battery for clinical trials. Schizophrenia Res. (2004) 72:1–3. 10.1016/j.schres.2004.09.006 15531401

[23] Kay SR Fiszbein A Opler LA. The positive and negative syndrome scale (PANSS) for schizophrenia. Schizophrenia Bull. (1987) 13:261–76. 10.1093/schbul/13.2.261 3616518

[24] Guy W. ECDEU assessment manual for psychopharmacology. U. S. Department of Health, Education, and Welfare, Public Health Service. (1976). 10.1037/e591322011-001

[25] Brown D Nakagome K Cordes J Brenner R Gründer G Keefe RSE. Evaluation of the efficacy, safety, and tolerability of BI 409306, a novel phosphodiesterase 9 inhibitor, in cognitive impairment in schizophrenia: a randomized, double-blind, placebo-controlled, phase ii trial. Schizophr Bull. (2019) 45:350–9. 10.1093/schbul/sby049 29718385 PMC6403090

[26] Green JP. Histamine and the nervous system. Fed Proc. (1964) 23:1095–102. 14209805

[27] Passani MB Blandina P. Histamine receptors in the CNS as targets for therapeutic intervention. Trends Pharmacol Sci. (2011) 32:242–9. 10.1016/j.tips.2011.01.003 21324537

[28] Medhurst AD Atkins AR Beresford IJ Brackenborough K Briggs MA Calver AR. GSK189254, a novel H3 receptor antagonist that binds to histamine H3 receptors in Alzheimer's disease brain and improves cognitive performance in preclinical models. J Pharmacol Exp Ther. (2007) 321:1032–45. 10.1124/jpet.107.120311 17327487

[29] Fox GB Esbenshade TA Pan JB Radek RJ Krueger KM Yao BB. Pharmacological properties of ABT-239 [4-(2-{2-[(2R)-2-Methylpyrrolidinyl]ethyl}-benzofuran-5-yl)benzonitrile]: II. Neurophysiological characterization and broad preclinical efficacy in cognition and schizophrenia of a potent and selective histamine H3 receptor antagonist. J Pharmacol Exp Ther. (2005) 313:176–90. 10.1124/jpet.104.078402 15608077

[30] Berthier ML. Poststroke aphasia: epidemiology, pathophysiology and treatment. Drugs Aging. (2005) 22:163–82. 10.2165/00002512-200522020-00006 15733022

[31] Kimberg DY D'Esposito M Farah MJ. Effects of bromocriptine on human subjects depend on working memory capacity. Neuroreport. (1997) 8:3581–5. 10.1097/00001756-199711100-00032 9427330

[32] McDowell S. Differential effect of a dopaminergic agonist on prefrontal function in traumatic brain injury patients. Brain. (1998) 121:1155–64. 10.1093/brain/121.6.1155 9648550

[33] Takahashi H Kato M Hayashi M Okubo Y Takano A Ito H. Memory and frontal lobe functions; possible relations with dopamine D2 receptors in the hippocampus. Neuroimage. (2007) 34:1643–9. 10.1016/j.neuroimage.2006.11.008 17174573

[34] Jackson CR Ruan G-X Aseem F Abey J Gamble K Stanwood G. Retinal dopamine mediates multiple dimensions of light-adapted vision. J Neurosci. (2012) 32:9359–68. 10.1523/JNEUROSCI.0711-12.2012 22764243 PMC3400466

[35] Egan MF Zhao X Gottwald R Harper-Mozley L Zhang Y Snavely D. Randomized crossover study of the histamine H3 inverse agonist MK-0249 for the treatment of cognitive impairment in patients with schizophrenia. Schizophr Res. (2013) 146:224–30. 10.1016/j.schres.2013.02.030 23523692

[36] Ellenbroek BA Ghiabi B. Do histamine receptor 3 antagonists have a place in the therapy for schizophrenia? Curr Pharm Des. (2015) 21:3760–70. 10.2174/1381612821666150605105325 26044979

[37] Barak N Beck Y Albeck JH. Betahistine decreases olanzapine-induced weight gain and somnolence in humans. J Psychopharmacol. (2016) 30:237–41. 10.1177/0269881115626349 26839321

[38] Lezius F Adrion C Mansmann U Jahn K Strupp M. High-dosage betahistine dihydrochloride between 288 and 480 mg/day in patients with severe Menière's disease: a case series. Eur Arch Otorhinolaryngol. (2011) 268:1237–40. 10.1007/s00405-011-1647-2 21626121

