# Supplementary material for: High-Dose Betahistine Improves Cognitive Function in Patients With Schizophrenia: A Randomized Double-Blind Placebo-Controlled Trial
Source: Front Psychiatry. 2021 Nov 1;12:762656. doi: 10.3389/fpsyt.2021.762656 (PMC8591287; doi:10.3389/fpsyt.2021.762656)
Supplement: Supplementary file 1 [file Data_Sheet_1.docx]

Supplementary Material

**About pilot study and sample size calculation**

Before the start of this RCT, we first conducted a single-arm pilot study (not yet published). This pilot study has been registered on the website of China Clinical Trial Center (ChiCTR2100048848). The pilot study was designed to treat schizophrenia patients (n=31) taking olanzapine with betahistine for 12 weeks. The cognitive function of the patients was tested at baseline and 12 weeks. The results of the pilot study showed that the MCCB composite score of patients with schizophrenia had changed by an average of 6.7 points (SD, 6). The results of Brown et al. found that the average change in the MCCB composite score of patients with schizophrenia after 12 weeks of treatment with placebo was 2.5 points.(1) Taking into account the drop-off rate of about 20% during the experiment, we used the PASS software to calculate that each group requires at least 42 people.

1. Brown D, Nakagome K, Cordes J, Brenner R, Gründer G, Keefe RSE, et al. Evaluation of the efficacy, safety, and tolerability of BI 409306, a novel phosphodiesterase 9 inhibitor, in cognitive impairment in schizophrenia: a randomized, double-blind, placebo-controlled, phase ii trial. Schizophr Bull (2019) 45:350–9. doi:10.1093/schbul/sby049

eTable 1

Adverse effects by treatment between groups.

| Adverse event | Betahistine group | Placebo group | χ^2^ | p-value |
| --- | --- | --- | --- | --- |
| Abnormal hematology (no., %) | 18 (40.00%) | 18 (40.91%) | 0.00 | 0.93 |
| Abnormal liver findings (no., %) | 9 (20.00%) | 9 (20.45%) | 0.00 | 0.96 |
| Tremor (no., %) | 8 (17.78%) | 5 (11.36%) | 0.73 | 0.39 |
| Dry mouth (no., %) | 1 (2.22%) | 1 (2.27%) | 0.00 | 0.99 |
| Constipation (no., %) | 7 (15.56%) | 9 (20.45%) | 0.36 | 0.55 |
| Nausea (no., %) | 3 (6.67%) | 3 (6.82%) | 0.00 | 0.98 |
| Syncope/dizziness (no., %) | 0 (0.00%) | 1 (2.27%) | 1.03 | 0.31 |
| Tachycardia (no., %) | 4 (8.89%) | 3 (6.82%) | 0.13 | 0.72 |
| Hypertension (no., %) | 1 (2.22%) | 1 (2.27%) | 0.00 | 0.99 |
| EKG abnormality (no., %) | 1 (2.22%) | 4 (9.09%) | 1.98 | 0.16 |
| Anorexia/decreased appetite (no., %) | 1 (2.22%) | 1 (2.27%) | 0.00 | 0.99 |
| Increased blood concentration of antipsychotics (no., %) | 1 (2.22%) | 0 (0.00%) | 0.99 | 0.32 |

- The p-values are derived from the chi-squared analysis.
